# Sm-Like Protein-Mediated RNA Metabolism Is Required for Heat Stress Tolerance in *Arabidopsis*

**DOI:** 10.3389/fpls.2016.01079

**Published:** 2016-07-21

**Authors:** Masanori Okamoto, Akihiro Matsui, Maho Tanaka, Taeko Morosawa, Junko Ishida, Kei Iida, Yoshiki Mochizuki, Tetsuro Toyoda, Motoaki Seki

**Affiliations:** ^1^Arid Land Research Center, Tottori UniversityHamasaka, Japan; ^2^RIKEN Center for Sustainable Resource ScienceYokohama, Japan; ^3^PRESTO, Japan Science and Technology AgencyKawaguchi, Japan; ^4^Graduate School of Medicine, Kyoto UniversityKyoto, Japan; ^5^RIKEN Center for Integrative Medical SciencesYokohama, Japan; ^6^RIKEN Advanced Center for Computing and CommunicationWako, Japan; ^7^Kihara Institute for Biological Research, Yokohama City UniversityYokohama, Japan; ^8^CREST, Japan Science and Technology AgencyKawaguchi, Japan

**Keywords:** arid region, heat stress, Sm-like protein, RNA metabolism, antisense RNA, non-coding RNA, aberrant transcriptions, *Arabidopsis*

## Abstract

Sm-like proteins play multiple functions in RNA metabolism, which is essential for biological processes such as stress responses in eukaryotes. The *Arabidopsis thaliana sad1* mutant has a mutation of sm-like protein 5 (LSM5) and shows impaired drought and salt stress tolerances. The *lsm5*/*sad1* mutant also showed hypersensitivity to heat stress. GFP-fused LSM5/SAD1 was localized in the nucleus under optimal growth conditions. After heat stress treatment, GFP-fused LSM5/SAD1 fluorescence was also observed as small cytoplasmic dots, in addition to nuclear localization. Whole genome transcriptome analysis revealed that many genes in *Arabidopsis* were drastically changed in response to heat stress. More heat-responsive genes were highly expressed in *lsm5*/*sad1* mutant at both 2 and 6 h after heat stress treatment. Additionally, intron-retained and capped transcripts accumulated in the *lsm5*/*sad1* mutant after heat stress treatment. In this study, we also identified non-*Arabidopsis* Genome Initiative transcripts that were expressed from unannotated regions. Most of these transcripts were antisense transcripts, and many capped non-AGI transcripts accumulated in the *lsm5*/*sad1* mutant during heat stress treatment. These results indicated that LSM5/SAD1 functions to degrade aberrant transcripts through appropriate mRNA splicing and decapping, and precise RNA metabolic machinery is required for heat stress tolerance.

## Introduction

Higher plants are sessile organisms that remain in the same habitat during their life, even if their surrounding environment changes to unfavorable conditions. Thus, plants have complex defense systems against various environmental stresses. For plants, heat stress is one of the major environmental stresses, and the atmospheric temperature can drastically increase depending on daily weather changes. To adapt to high temperatures, plants dynamically regulate the transcription levels of genes related to heat stress. Heat shock transcription factors (HSFs) are induced in response to heat stress and regulate the expression of heat shock proteins (HSPs) and ascorbate peroxidase (APX) (Panchuk et al., 2002; Wang et al., 2004; Kotak et al., 2007). HSPs function as molecular chaperones to maintain cellular homeostasis under both optimal and adverse growth conditions. There are several families of HPSs in plants. APX has an important role in scavenging heat stress-inducible reactive oxygen species (ROS) (Panchuk et al., 2002). It is well known that HSFs, HSP, and APX are responsible for heat stress tolerance.

Heat stress also influences post-transcriptional regulation of mRNA, such as pre-mRNA processing, mRNA stability, degradation, transport and localization (Floris et al., 2009). The molecular relationship between heat stress and RNA metabolic factors has been revealed from molecular studies of decapping complex and 5′-exoribonuclease (Maldonado-Bonilla, 2014). *Arabidopsis* decapping protein 2 (DCP2)/TRIDENT (TDT), but not DCP1, can remove m^7^GDP from the 5′ end of mRNA and binds to DCP1 and VARICOSE (VSC) (Xu et al., 2006). A complex of DCP1, DCP2 and VSC are involved in decapping *in vivo* (Xu et al., 2006; Goeres et al., 2007). On the other hand, *Arabidopsis* exoribonuclease 4 (XRN4) functions in the degradation of 5′ to 3′ uncapped mRNA (Kastenmayer and Green, 2000; Souret et al., 2004). DCP1, VSC, XRN4 co-localize to processing-bodies (P-bodies) in the cytoplasm (Xu et al., 2006; Goeres et al., 2007; Weber et al., 2008). Interestingly, DCP2/TDT is present throughout the cytoplasm under optimal growth conditions and then co-localizes to P-bodies with DCP1 in response to heat stress (Motomura et al., 2015). These observations suggest that drastic control is exerted over the mRNA degradation machinery under environmental changes.

In addition, Sm-like proteins (LSMs) function in multiple aspects of RNA metabolism. There are eight conserved LSM proteins in animals, yeast and plants (Wilusz and Wilusz, 2005; Parker and Sheth, 2007; Franks and Lykke-Andersen, 2008; Perea-Resa et al., 2012). The cytoplasmic LSM1-7 complex binds to oligoadenylated mRNA, promotes its decapping and is involved in its degradation. Therefore, *lsm1* and *lsm5* (also known as *sad1*) mutants accumulate capped mRNA, and increased RNA stability is observed in these mutants (Perea-Resa et al., 2012; Golisz et al., 2013). By contrast, the nuclear LSM2-8 complex binds to U6 small nuclear RNA and forms the core of the small nuclear ribonucleoprotein particle, which functions in pre-mRNA splicing (Beggs, 2005; Wilusz and Wilusz, 2005; Tharun, 2009). Indeed, the *lsm5/sad1* mutant accumulates unspliced mRNA precursors (Golisz et al., 2013; Cui et al., 2014). The cellular localizations of LSM3 and 4 change in response to heat stress, whereas LSM1 and LSM8 localize to cytoplasmic foci and the nucleus, respectively (Perea-Resa et al., 2012). Thus, the functions of LSMs, except for LSM1 and LSM8, are thought to change depending on environmental conditions.

In this study, to reveal the transcriptional regulation via LSM5/SAD1 under the high temperature, genome-wide transcriptome analysis in the *lsm5/sad1* mutant was performed during heat stress treatment. Unspliced and capped transcripts among heat stress-inducible genes accumulated in the *lsm5/sad1* mutant. Moreover, heat inducible aberrant antisense transcripts also accumulated and had capped transcripts in the *lsm5/sad1* mutant. These results indicated that LSM5/SAD1 contributes to the degradation of heat-stress inducible aberrant transcripts through appropriate mRNA splicing and decapping.

## Materials and Methods

### Plant Materials and Stress Condition

The wild-type and *lsm5/sad1* mutant of the C24 accession of *Arabidopsis thaliana* were used (Xiong et al., 2001). The *lsm5/sad1* (CS24935) mutant used in this study was obtained from the *Arabidopsis* Biological Resource Center (ABRC). Plants were grown in plastic plates (57 mm × 16 mm) containing 10 mL agar media (0.8% agar plate containing 1% sucrose, B5 vitamin and 2.5 mM MES (pH5.8)) under continuous light at 22°C. For the thermotolerance assay, agar plates with 5-day-old seedlings were transferred to high temperature conditions for 90 min and then returned to 22°C. The survival rate and chlorophyll content were determined after 9 days of growth. For the transcript and LSM5/SAD1 protein analyses in response to heat stress, agar plates including 10-day-old seedlings grown at 22°C were transferred to 37°C for 1, 2, 3, and 6 h, and seedlings were frozen immediately in liquid nitrogen after the stress treatments.

### Measurement of Chlorophyll Content

Chlorophyll from seedlings was extracted by using dimethylformamide. The absorbance values of extracts were obtained using a SmartSpec-3000 (Bio-Rad), and chlorophyll contents were calculated using a previously described formula (Chlorophyll a + b = 17.67 × (A_647_-A_750_)+ 7.12 × (A_664_-A_750_) (Porra et al., 1989).

### Reverse Transcription Polymerase Chain Reaction (RT-PCR) Analysis

Total RNA from 10-day-old seedlings was isolated using the ISOGEN reagent (NIPPON GENE) according to the manufacturer’s protocol. The RNA was purified using lithium chloride precipitation. Purified total RNA was treated with RQ1 RNase-Free DNase (Promega). For RT-PCR analysis, first-strand cDNA was synthesized using oligo(dT) primer and SuperScript^®^ III Reverse Transcriptase (Thermo Fisher Scientific). To analyze sense and antisense transcripts, total RNA was reverse-transcribed with sequence-specific reverse and forward primers, respectively, as previously described by (Matsui et al., 2008). PCR products were loaded on 1% high-resolution gels and visualized using ethidium bromide. The primer sets and PCR conditions are shown in Supplementary Table S5.

### Transgenic Plants

To generate transgenic plant expressing GFP-tagged LSM5/SAD1 under the control of the *LSM5/SAD1* promoter, the genomic sequence for *LSM5/SAD1* (*At5g48870*) containing a 2911bp region upstream of the start codon and excluding the stop codon was amplified by PCR using primers 5′-CACCGCGAATCATCGTCACTCTCAGTCG-3′ and 5′-TTCTCCATCTTCGGGAGACCCACCT-3′. The underlined sequence in the primer indicates the adaptor site for the pENTR/D-TOPO vector (Thermo Fisher Scientific). The PCR fragment was cloned into the vector and subsequently cloned into the binary vector pGWB3 (C-terminal GFP fusion) using the Gateway LR reaction system (Thermo Fisher Scientific). The resulting plasmid was electroporated into *Agrobacterium tumefaciens* strain GV3101 and introduced into *lsm5/sad1* mutant plants by the floral-dipping method. To generate transgenic plants constitutively expressing GFP, the *GFP* sequence, with a stop codon, was cloned into the binary vector pGWB2 (under control of the 35S promoter). The plasmid was introduced into the wild-type as described above. Binary vectors pGWB2 and pGWB3 were reported previously (Nakagawa et al., 2007). T1 transgenic plants were selected with kanamycin and hygromycin. Approximately 20 independent transgenic lines were grown on soil. Homozygous T3 lines were used for GFP observation and detection of the SAD-GFP fusion protein.

### Immunoblot Analysis

Seedlings were ground in liquid nitrogen and extracted in a buffer containing 100 mM Tris-HCl (pH8.0), 0.5% SDS, 10% glycerol and 2% β-mercaptoethanol. Extracts were heated for 5 min at 95°C, and clear lysates were obtained after centrifugation. The amount of extracted protein was determined using a Quick Start^TM^ Bradford assay (Bio-Rad). After the extracted proteins were heated with SDS sample buffer, 20 μg of total protein was subjected to SDS-PAGE. To detect the LSM5/SAD1-GFP fusion protein, polyclonal rabbit anti-GFP antibodies (Thermo Fisher Scientific, A11122) and horseradish peroxidase-conjugated antibodies against rabbit IgG (GE Healthcare) were used. The ECL western-blotting detection system was used to detect the chemiluminescent signal (GE Healthcare) as described previously (Myouga et al., 2008).

### Microscopy Analysis

Transgenic seedlings grown at 22°C were subjected to 37°C for 6 h. GFP fluorescence was observed in the hypocotyl tissues before and after heat stress treatments using confocal laser scanning microscopy (LSM510 Meta; Zeiss). The excitation and emission wavelengths for GFP were 488 nm and 505–530 nm, respectively. Chlorophyll self-fluorescence was detected using a 560 nm long pass filter.

### Whole-Genome Tiling Array Analysis

The *Arabidopsis* whole-genome tiling array set (1.0 F array and 1.0 R array, Affymetrix) was used in this study, and three independent biological replicates were performed for each strand array. Probe synthesis, array hybridization and data scanning were performed as described in Matsui et al. (2008). *Arabidopsis* annotation information from TAIR8 was mapped to the whole-genome tiling array. Tiling array data analysis was carried out essentially as described previously (Matsui et al., 2008; Okamoto et al., 2010). The expressed *Arabidopsis* Genome Initiative (AGI) code genes and non-AGI transcriptional units (TUs) were detected using the ARTADE program (*P* initial value < 10^-8^) (Toyoda and Shinozaki, 2005). Furthermore, non-AGI TUs were compared with the information in TAIR10 (Supplementary Table S3). To identify the differentially expressed AGI code genes and non-AGI TUs between the wild-type and *lsm5/sad1* mutant during heat stress, significant differences were judged using the Mann-Whitney *U* test (false discovery rate, α = 0.05) using the all probes (5.8 million perfect match and 5.8 million mismatch probes) as described in (Storey and Tibshirani, 2003; Matsui et al., 2008). To conduct hierarchical clustering analysis, tiling array data were entered into GENESPRING (Ver. 7.3, Agilent Technologies). The *Arabidopsis* tiling array data used in this study is available at GEO^1^ under the accession number GSE44620.

### Rapid Amplification of cDNA Ends (RACE) Analysis of Capped mRNAs

To determine whether accumulated transcripts in the *lsm5/sad1* mutant are capped, RNA ligase-mediated RACE was performed using the GeneRacer^TM^ Kit (Thermo Fisher Scientific) as described previously (Kurihara et al., 2009; Okamoto and Seki, 2011). Total RNA was treated with calf intestinal phosphatase and subsequently treated with tobacco acid pyrophosphatase (TAP) to remove the 5′ cap structure from intact mRNA. GeneRacer^TM^ RNA Oligo (Thermo Fisher Scientific) was ligated to the 5′ end of the mRNA using T4 RNA ligase. The ligated RNA was reverse-transcribed to synthesize first-strand cDNA using SuperScript III RT with oligo(dT) primers. To detect the GeneRacer^TM^-tagged cDNA, 5′-race PCR was performed with the GeneRacer^TM^ 5′ forward primer and a gene specific reverse primer. The second PCR was performed with the GeneRacer^TM^ 5′ nested primer and a gene specific reverse primer, using first PCR products as templates. Visualization of 5′-RACE products was performed as described in section 2.3. The primer information is shown in Supplementary Table S5.

## Results

### *Arabidopsis* LSM5/SAD1 Is Required for Heat Stress Tolerance

**FIGURE 1:**
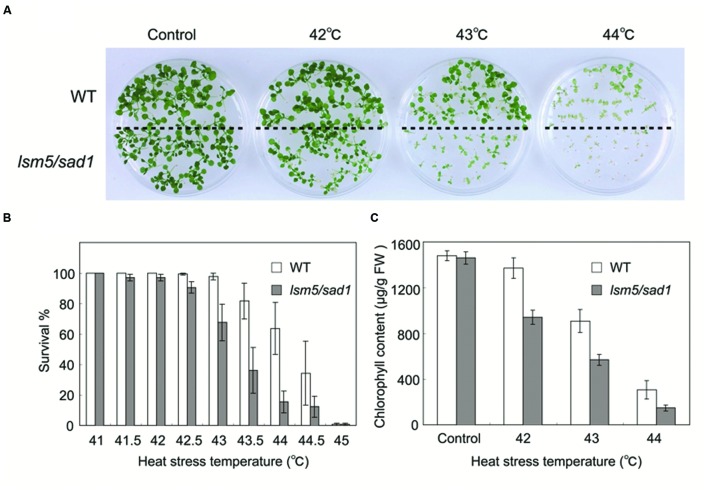
**Thermotolerance of the *lsm5/sad1* mutant after heat stress treatment.** **(A)** Photographs of wild-type and *lsm5/sad1* mutants after heat stress treatment. The survival rate **(B)** and chlorophyll content **(C)** of heat-treated wild-type and *lsm5/sad1* mutant after heat stress at various temperatures. Five-day-old seedlings of the wild-type and *lsm5/sad1* mutant were subjected to various temperatures for 90 min, and then returned to 22°C under the light, and photographs, survival rate and chlorophyll content were obtained after 9-days of growth. **(B**,**C)** The values are means ± SEM of the result from five plates (∼25 plants per plate) of individual lines.

The *Arabidopsis lsm5/sad1* mutant was originally isolated as a hypersensitive mutant to salt and drought (Xiong et al., 2001). To investigate the relationship between LSM5/SAD1 and heat stress, we conducted heat tolerance assays using the *lsm5/sad1* mutant. The wild-type and *lsm5/sad1* mutant were grown on agar plates at 22°C for 5 days and then exposed at various heat temperatures for 90 min before being returned to 22°C. We photographed the plants’ phenotypes after 9 days of recovery. Seedling growth of the *lsm5/sad1* mutant was inhibited by heat stress at 42°C and began to die at a temperature higher than 43°C (**Figures 1A,B**). This heat stress tolerance of the *lsm5/sad1* mutant was weaker than that of the wild-type. We then measured the chlorophyll content of the *lsm5/sad1* mutant after heat stress treatment, because heat stress bleaches the chlorophyll pigment of plants’ green tissues. Consistent with the plant growth and survival rate results, the chlorophyll content of the *lsm5/sad1* mutant was reduced compared with the wild-type (**Figure 1C**). This impaired thermotolerance of the *lsm5/sad1* mutant was complemented by introducing the promoter_SAD1_::SAD1-sGFP construct, which restored wild-type levels of thermotolerance (**Supplementary Figures S1A-C**). These results indicated that SAD1 is required for heat stress tolerance.

### Expression Pattern and Protein Localization of LSM5/SAD1 in Response to Heat Stress

**FIGURE 2:**
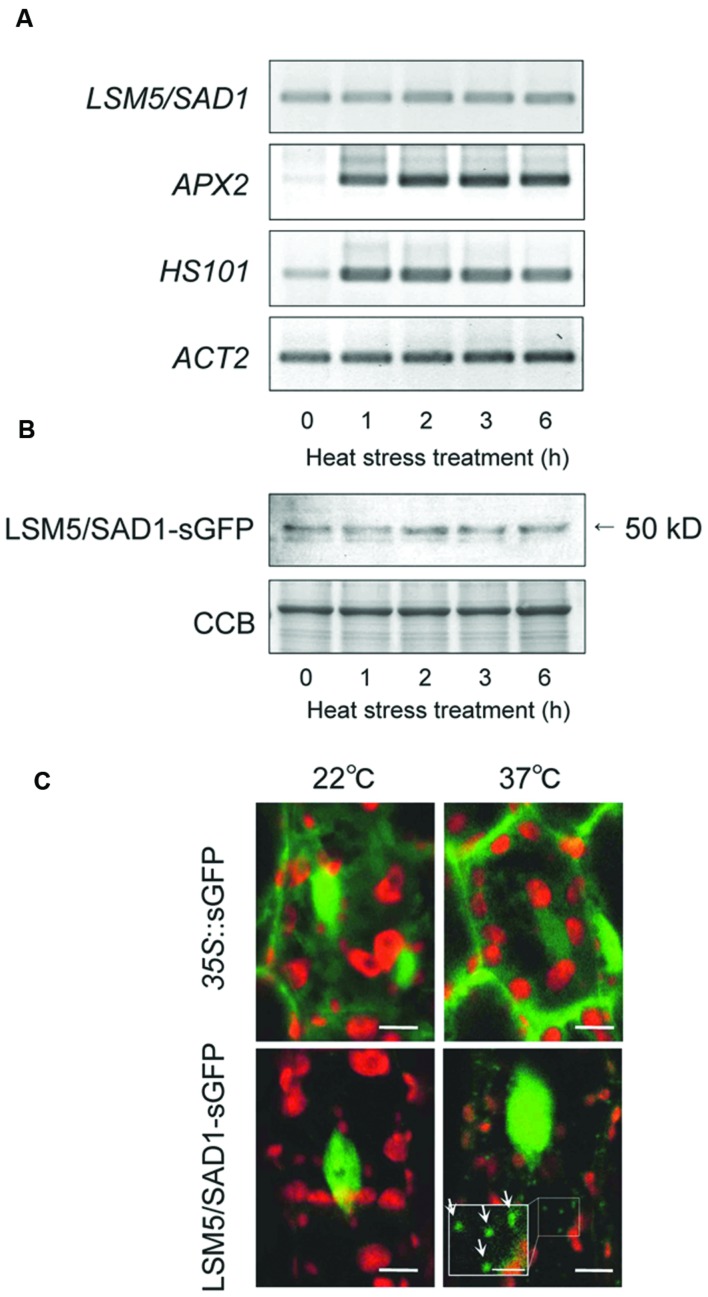
**Transcripts, protein levels and subcellular protein localization of *LSM5/SAD1* during heat stress.** **(A)** Expression patterns of *LSM5/SAD1* and heat responsive marker genes, *APX2* and *HSP101*, during heat stress in wild-type plants. *ACT2* was used as an internal control. **(B)** Expression patterns of LSM5/SAD1-sGFP protein during heat stress. Transgenic plants expressing the LSM5/SAD1-sGFP fusion protein driven by the *LSM5/SAD1* promoter were generated in the *lsm5/sad1* mutant. This pro_LSM5/SAD1_::LSM5/SAD1-sGFP construct complimented the phenotype of the *lsm5/sad1* mutant. Twenty micrograms of total protein was loaded in each lane and immunologically detected using an anti-GFP antibody. Coomassie-stained portion (Cms) of gel is shown as a loading control. **(A**,**B)** 10-day-old seedlings were incubated at 37°C for 0, 1, 2, 3, and 6 h, and total mRNA and protein were extracted. **(C)** Confocal microscope images of the sGFP fluorescence of the *pro_LSM5/SAD1_*::LSM5/SAD1-sGFP transgenic *lsm5/sad1* plants and *35S*::sGFP transgenic plants. The hypocotyl tissues were observed under the microscope before and after incubation at 37°C for 6 h. Green and red fluorescences indicate the sGFP signal and autofluorescence from the chloroplast, respectively. White arrows indicate cytoplasmic dots of LSM5/SAD1-sGFP fluorescence. Bars = 5 μm, whereas closed-up bar = 2.5 μm.

To examine how LSM5/SAD1 contributes to heat stress tolerance, we examined *LSM5/SAD1* expression during heat stress. Although heat responsive marker genes, *APX2* and *HSP101*, were upregulated by heat stress, *LSM5/SAD1* expression did not change significantly during heat stress (**Figure 2A**). We then examined LSM5/SAD1 protein levels using transgenic plants expressing the LSM5/SAD1-GFP fusion protein driven by the LSM5/SAD1 promoter. This pro_LSM5/SAD1_::LSM5/SAD1-sGFP construct complemented the phenotype of the *lsm5/sad1*mutant (**Supplementary Figure S1**), suggesting that the LSM5/SAD1-GFP fusion protein functions like the native LSM5/SAD1 protein *in vivo*. Anti-GFP antibodies recognized a single protein band of 50 kDa, and the level of the detected LSM5/SAD1-sGFP fusion protein remained constant during heat stress (**Figure 2B**). Furthermore, we examined the intracellular localization of LSM5/SAD1-GFP fusion protein, because a subset of Sm-like proteins in *Arabidopsis* forms cytoplasmic foci in response to stress (Perea-Resa et al., 2012). GFP fluorescence in pro_LSM5/SAD1_::LSM5/SAD1-GFP transgenic plants was mainly observed in the nucleus under optimal growth conditions. After heat stress treatment, the nuclear localization of LSM5/SAD1-GFP did not change. However, LSM5/SAD1-GFP fluorescence was also observed as small cytoplasmic dots (**Figure 2C**). By contrast, GFP fluorescence in *35S*::GFP transgenic plants was observed in both the cytoplasm and the nucleus before and after heat stress (**Figure 2C**). These results indicated that the LSM5/SAD1 protein mainly functions in nucleus, but the distribution of a fraction of it is dynamically changed in response to heat stress, similar to LSM3 and LSM4 (Perea-Resa et al., 2012).

### Genome-Wide Analysis of LSM5/SAD1-Regulated Genes during Heat Stress

**FIGURE 3:**
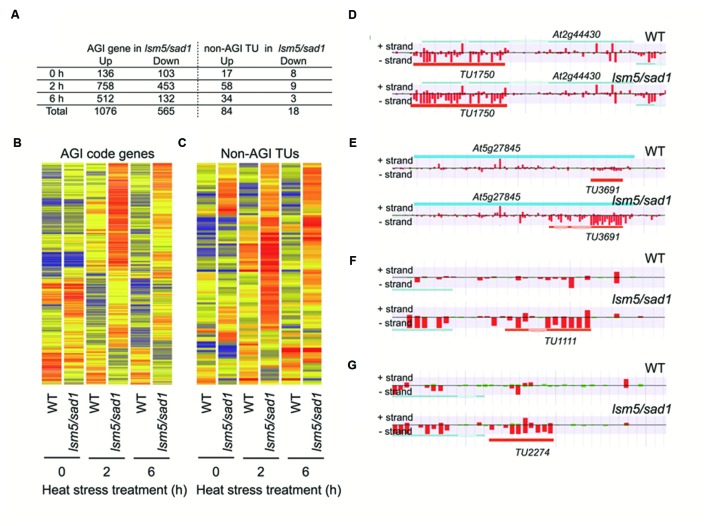
***Arabidopsis* Genome Initiative (AGI) genes and non-AGI transcriptional units (TUs) that are differentially expressed between the wild-type and the *lsm5/sad1* mutant during heat stress.** **(A)** Number of AGI code genes and non-AGI TUs that were upregulated and downregulated in the *lsm5/sad1* mutant during heat stress. AGI genes and non-AGI TUs that were differentially expressed between the wild-type and the *lsm5/sad1* mutant, as judged by the Mann-Whitney *U* test (false discovery rate, α = 0.05 ), were further selected using an expression ratio cut-off of twofold higher or lower. Hierarchical clustering analysis of differentially expressed 1641 AGI code genes **(B)** and 102 non-AGI TUs **(C)** between the wild-type and the *lsm5/sad1* mutant during heat stress. Colored bars indicate relative expression levels. **(D,E)** Examples of antisense non-AGI TUs upregulated in the *lsm5/sad1* mutant. **(F**,**G)** Examples of intergenic non-AGI TUs upregulated in the *lsm5/sad1* mutant. Tiling array data after incubation at 37°C for 6 h shown in **(D**-**G)**. In **(D**-**G)**, blue and orange horizontal bars indicate AGI code genes and predictive non-AGI TU, respectively. Red and green bars indicate the signal intensity of the probes (red > 400, green < 400).

To reveal the global transcription profiles regulated by LSM5/SAD1, we carried out whole-genome transcriptome analysis of the *lsm5/sad1* mutant using strand specific tiling arrays. Under the optimal growth conditions (non-heat stressed), there were 136 upregulated and 105 downregulated AGI genes in the *lsm5/sad1* mutant compared with AGI-code genes expressed in the wild-type (**Figure 3A**, Supplementary Tables S1 and S2). In contrast, 758 and 512 upregulated and 453 and 132 downregulated AGI-code genes in the *lsm5/sad1* mutant were identified compared with AGI-code genes expressed in the wild-type at 2 and 6 h after heat stress treatment, respectively (**Figure 3A**, Supplementary Tables S1 and S2). During heat stress, there were more up and downregulated AGI-code genes at 2 h after heat stress treatment than at 6 h. This result was probably because the *lsm5/sad1* mutant is hypersensitive to heat stress. Additionally, 243 upregulated and 72 downregulated AGI-code genes overlapped among the upregulated and downregulated AGI-code genes during heat stress treatment. Interestingly, many genes related to heat stress, such as HSF, and HSP, were included among the upregulated AGI genes, but not the downregulated AGI genes after heat stress treatment, despite the *lsm5/sad1* mutant showing a weak phenotype for heat tolerance (Supplementary Tables S1 and S2).

**FIGURE 4:**
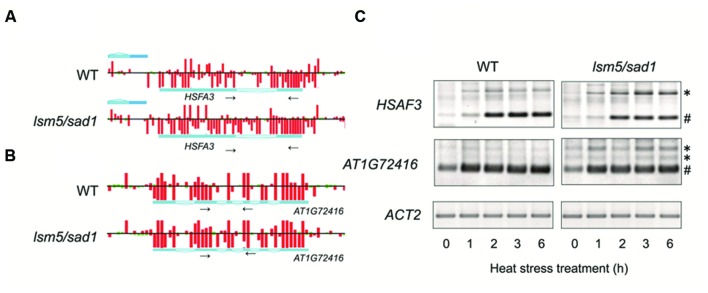
**Detection of transcripts improperly spliced in the *lsm5/sad1* mutant.** Example of the impaired splicing of *HSFA3* **(A)** and *AT1G72416* **(B)** in the *lsm5/sad1* mutant, as detected by a tiling array. Pale blue regions in the horizontal bars indicate intron in transcripts. Red and green bars indicate the signal intensity of the probes (red > 400, green < 400). Tiling array data after incubation at 37°C for 6 h shown in **(A,B)**. Small black arrows indicate primers used for RT-PCR analysis in **(C)**, and these regions detected retained an intron in the *lsm5/sad1* mutant, as detected by a tiling array. **(C)** Semi-quantitative RT-PCR analysis to detect unspliced *HSFA3* and *AT1G72416* transcripts in the *lsm5/sad1* mutant. Spliced and unspliced PCR products are labeled as # and ^∗^, respectively. Ten-day-old seedlings were incubated at 37°C for 0, 1, 2, 3, and 6 h.

Unspliced mRNA precursors accumulated in the *lsm5/sad1* mutant (Golisz et al., 2013; Cui et al., 2014). Therefore, we focused on the expression of genes related to heat stress and whether aberrant splicing occurred in the *lsm5/sad1* mutant. We found that strong expression of *HSFA3*, which encodes a heat-shock transcription factor, could be observed in the intron region on the tiling array in the *lsm5/sad1* mutant, but not in the wild-type at 6 h after heat stress treatment (**Figure 4A**). To assess intron expression of *HSFA3* in the tiling array, we used RT-PCR analysis. Unspliced *HSFA3* transcripts were detected in the *lsm5/sad1* mutant after heat stress treatment, and the generated band was stronger than in the wild-type (**Figure 4A**). In contrast to the unspliced transcripts, mature *HSFA3* transcripts in *lsm5/sad1* mutant were reduced compared with the wild-type during heat stress treatment (**Figure 4C**). HSFA3 is a key transcription factor for heat stress tolerance (Schramm et al., 2008; Yoshida et al., 2008). Additionally, we found aberrant splicing of *AT1G72416*, which encodes a HSP, in the *lsm5/sad1* mutant during heat stress treatment (**Figures 4B,C**). Therefore, there aberrant splicing in the *lsm5/sad1* mutant could be regarded as one of the causes reducing heat stress tolerance.

**FIGURE 5:**
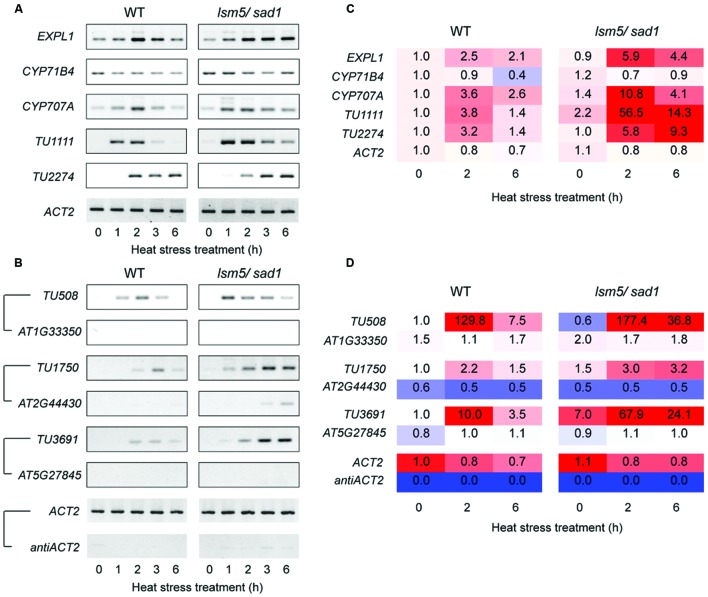
**Semi-quantitative RT-PCR analysis for differentially expressed transcripts between the wild-type and the *lsm5/sad1* mutant during heat stress.** **(A)** Expression patterns of intergenic-type AGI code genes and non-AGI TUs that were upregulated in the *lsm5/sad1* mutant. First-strand cDNA synthesis was performed using oligo(dT) primers, and then PCR was performed using transcript-specific primers. **(B)** Expression patterns of antisense non-AGI TUs that were upregulated in the *lsm5/sad1* mutant. First-strand cDNA synthesis was performed using strand-specific primers for transcripts, and then PCR was performed using transcript-specific primers. Pairs of antisense/sense transcripts are as follows: T*U508/AT1G33350*, *TU1750/AT2G44430*, *TU3691/AT5G27845*, and *antiACT2/ACT2*. *ACT2* and *antiACT2* were used as internal controls. Ten-day-old seedlings were incubated at 37°C for 0, 1, 2, 3, and 6 h. **(C,D)** The fold change values were calculated from transcriptional expression intensities on tiling array data (Supplementary Tables S1 and S3).

**FIGURE 6:**
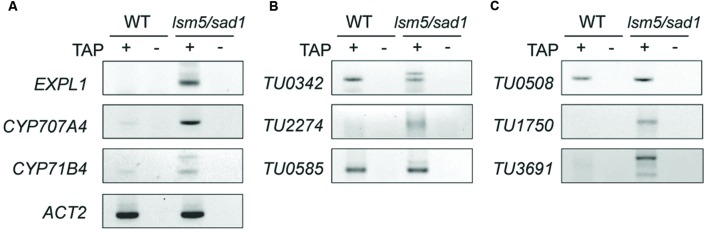
**Detection of capped transcripts abnormally accumulated in the *lsm5/sad1* mutant.** RACE-PCR analysis of AGI-code genes **(A)**, intergenic non-AGI TUs **(B)**, and antisense type of non-AGI TUs **(C)**. Capped transcripts were examined by RNA ligase-mediated RACE. Total RNA was incubated with calf intestinal phosphatase to remove the 5′ phosphates, and an RNA adapter was ligated to the 5′ end of the transcript after a decapping reaction by tobacco acid pyrophosphatase (TAP) treatment. (+) and (-) indicate with and without TAP treatments, respectively. After the RT-reaction, using oligo(dT) primers, the first PCR was performed using an RNA adapter and transcript-specific primer sets, and then nested PCR was performed using a nested RNA adapter and first transcript-specific primer sets. TAP (-) indicates a negative control for RACE-PCR. Total RNA was extracted from heat-treated samples.

DCP2/TDT and VSC are involved in RNA decapping, and this event is required before RNA degradation (Xu et al., 2006; Goeres et al., 2007). In addition, the LSM1-7 complex also contributes to RNA decapping (Tharun and Parker, 2001; Perea-Resa et al., 2012; Golisz et al., 2013). Target genes of DCP2/TDT and VSC are reported as *EXPL1*, *CYP71B4*, and *CYP707A4* (Goeres et al., 2007). To test whether these genes are also target genes of LSM5/SAD1, we checked expression levels of these genes. Tiling array and RT-PCR analyses revealed expression levels of *EXPL1*, *CYP71B4*, and *CYP707A4* in the *lsm5/sad1* mutant were higher compared with the wild-type (**Figures 3A,B** and **5A,C, ** Supplementary Table S2). In the case of *EXPL1* and *CYP707A4*, intron retaining transcripts were also observed in the *lsm5/sad1* mutant during heat stress treatment (**Figure 5A**). To reveal whether *EXPL1*, *CYP71B4*, and *CYP707A4* transcripts in the *lsm5/sad1* mutant retain their 5′ cap, we performed 5′-RACE PCR using a GeneRacer kit, which is an RNA ligase-mediated RACE method. Ligation products were not detected without TAP treatment as negative control (**Figure 6A**). In the TAP treatments, significant 5′-RACE products for *EXPL1*, *CYP71B4*, and *CYP707A4* were detected in the *lsm5/sad1* mutant, whereas none were detected in the wild-type (**Figure 6A**). By contrast, 5′-RACE products for *ACT2* were detected at similar levels between the wild-type and the *lsm5/sad1* mutant (**Figure 6A**). This result indicated that LSM5/SAD1 targets specific transcripts. Interestingly, multiple PCR bands for *EXPL1* and *CYP71B4* were detected in the *lsm5/sad1* mutant (**Figure 6A**). It is likely that transcripts of *EXPL1* and *CYP71B4* in the *lsm5/sad1* mutant might retain the cap on different locations on their 5′ terminus.

### LSM5/SAD1 Targets Aberrant Transcripts

In this study, we identified 4,228 non-AGI TUs that were not registered in The *Arabidopsis* Information Resource (TAIR) (Supplementary Table S3). Among them, 3,589 non-AGI TUs (84%) were categorized as antisense transcripts (Supplementary Table S3). Eighty-four upregulated and 18 downregulated non-AGI TUs were identified in the *sad1* mutant compared with non-AGI TUs expressed in the wild-type under both optimal growth conditions and heat stress conditions (**Figures 3A,B**; Supplementary Table S4). During heat stress treatment, 58 and 34 upregulated and nine and three downregulated non-AGI TUs in the *lsm5/sad1* mutant were identified compared with non-AGI TUs expressed in the wild-type at 2 and 6 h after heat stress treatment, respectively (Supplementary Table S3). Among the 84 upregulated non-AGI TUs in the *lsm5/sad1* mutant, 62 non-AGI TUs (74%) were defined as antisense transcripts, whereas 22 (26%) were expressed from intergenic regions (**Figures 3D–G**, Supplementary Table S4). Contrastingly, among the downregulated non-AGI TUs in *lsm5/sad1*, 7 and 11 were categorized as antisense and intergenic types, respectively (Supplemental Table S4). Indeed, the expressions of *TU1111* and *TU2227* were detected using RT-PCR analysis, and their expression intensities in the *lsm5/sad1* mutant tended to be stronger than those in the wild-type at 6 h after heat stress treatment, which was consistent with the tiling array data (**Figures 5A,C**, Supplementary Table S3). Additionally, we also confirmed antisense transcripts that were expressed on the opposite strand to AGI-code genes using strand specific RT-PCR analysis. *AT1G33350*, *At2G44430*, and *AT5G27845* encode a Pentatricopeptide repeat (PPR) superfamily protein, MYB-like DNA-binding bromodomain-containing protein and non-LTR retrotransposon family, respectively, and these show low expression levels in developmental stages and under various stress treatments (**Figure 5B**; Winter et al., 2007; Hanada et al., 2013). By contrast, expressions of *TU508*, *TU11750* and *TU3691* increased during heat stress, and their expression levels in *lsm5/sad1* were stronger than in the wild-type (**Figure 5B**). These expression patterns were similar to those observed in the tiling array analysis (**Figure 5D**, Supplementary Tables S1 and S3). These results indicated that non-AGI TUs identified by the strand specific tiling array were truly expressed in *Arabidopsis*.

To test whether the highly expressed non-AGI TUs in *lsm5/sad1* mutant retained their 5′ caps as well as AGI genes, we examined cap accumulation using a GeneRacer kit. In case of intergenic non-AGI TUs, the 5′-RACE product for *TU2274* was detected in only the *lsm5/sad1* mutant (**Figure 6B**). Although PCR products for *TU0342* and *TU0585* were detected in both the wild-type and the *lsm5/sad1* mutant, their multiple bands were observed only in the *lsm5/sad1* mutant (**Figure 6B**). Antisense non-AGI TUs in the *lsm5/sad1* mutant showed similar trends to AGI genes and intergenic non-AGI TUs (**Figure 6C**). These results suggested that capped antisense non-AGI TUs accumulate in the *lsm5/sad1* mutant.

## Discussion

In this study, we conducted comprehensive transcriptome analysis to understand the molecular function of the LSM5/SAD1 protein during heat stress. LSM5/SAD1 has multiple functions for RNA splicing, decapping, and degradation of heat-stress inducible aberrant antisense transcripts.

We observed that unspliced *HSFA3* transcripts accumulated in *lsm5/sad1*, whereas the amount of mature *HSFA3* mRNAs in *lsm5/sad1* mutant was reduced compared with the wild-type during heat stress treatment. HSFA3 functions as a key transcription factor to induce HSPs (Schramm et al., 2008; Yoshida et al., 2008). Thus, the reduction of the mature *HSFA3* mRNA in the *lsm5/sad1* mutant might be contributed to the weak phenotype for heat stress tolerance. In addition, unspliced transcripts of *HSP*, *EXPL1*, and *CYP707A4* were observed in the *lsm5/sad1* mutant during heat stress treatment (**Figure 4** and 5). Abnormal transcripts with retained introns were detected in *lsm4* and *lsm5/sad1* mutants under salt stress (Zhang et al., 2011; Cui et al., 2014). Interestingly, genes with abnormal splicing in salt-treated *lsm5/sad1* mutants are closely associated with stress responsive genes (Zhang et al., 2011; Cui et al., 2014). Therefore, the LSM5/SAD1 protein is thought to be required to control the splicing of stress-responsive genes under stress condition. Contrastingly, the *Arabidopsis root initiation defective1* (*rid1*) mutant shows a weak phenotype under heat stress, and the corresponding gene in this mutant encodes a DEAH-box RNA helicase, which is involved in pre-mRNA splicing (Ohtani et al., 2013). Similar to the *lsm4* and *lsm5/sad1* mutants, the *rid1* mutant exhibits reduced efficiency of pre-mRNA splicing under heat stress conditions (Ohtani et al., 2013). Taken together, accurate splicing of stress responsive genes by the spliceosomal complex with LSM proteins might be essential for heat stress tolerance in plants.

*Arabidopsis* LSM5/SAD1-GFP signals were detected in nucleus under both the 22°C and 37°C conditions, however, their signals were also observed in the cytoplasmic region after heat stress treatment (**Figure 2C**). LSM3 and LSM4 also show similar localization patterns to the LSM5/SAD1 protein at 22°C and 37°C (Perea-Resa et al., 2012). The LSM1-7 complex contributes to RNA decapping in eukaryotes (Tharun and Parker, 2001; Perea-Resa et al., 2012; Golisz et al., 2013). Therefore, LSM5/SAD1 might be involved in RNA decapping, because LSM5/SAD1 is a component of the LSM1-7 complex. Indeed, *EXPL1*, *CYP71B*, and *CYP707A4* mRNAs accumulated as capped mRNA in the *lsm5/sad1* mutant (**Figure 5A**, Supplementary Table S1). Consistent with our results, quantitative real-time RT-PCR analysis after immunoprecipitation using anti-cap antibodies from total RNAs revealed that the increased mRNA stabilization in the *lsm5/sad1* mutant resulted from a defect in decapping (Golisz et al., 2013). Interestingly, *EXPL1*, *CYP71B*, and *CYP707A4* mRNAs accumulated in *dcp2/tdt* mutants (Goeres et al., 2007). Thus, it is possible that a subset of target genes of the decapping complex might overlap with that of the LSM1-7 complex. Our results suggested that LSM5/SAD1 contributes to decapping of heat-inducible transcripts that are required for RNA degradation. However, it is noteworthy that HSFs in the *lsm5/sad1* mutant accumulated at 2 h after heat stress treatment (Supplementary Table S2). Thus, it is possible that some HSFs might enhance the transcriptional levels of AGI-code genes and non-AGI TUs during heat stress. Therefore, accumulated transcripts in the *lsm5/sad1* mutant during heat stress treatment could have resulted from both enhancement of transcription and repression of RNA degradation. Transcriptome analysis with transcription inhibitors would be useful to dissect the multiple effects on the accumulation of transcripts in the *lsm5/sad1* mutant.

Large-scale transcriptome analyses using strand specific tiling arrays and next-generation RNA sequencings have identified many naturally occurring antisense transcripts (Henz et al., 2007; Matsui et al., 2008; Kurihara et al., 2009; Okamoto et al., 2010; Liu et al., 2012, Li et al., 2013). Most antisense transcripts are classified as non-coding transcripts (Matsui et al., 2008; Kurihara et al., 2009; Okamoto et al., 2010). Therefore, these tend to be targeted by the nonsense-mediated mRNA decay pathway (Kurihara et al., 2009). Heat-inducible antisense transcripts in the *lsm5/sad1* mutant accumulated as capped transcripts, whereas the expressions of these transcripts in the wild-type were relatively low (**Figure 6**). These results indicated that LSM5/SAD1 might be involved in the degradation of heat stress-inducible aberrant antisense transcripts through promoting their decapping. By contrast, naturally occurring antisense transcripts have various functions (Pelechano and Steinmetz, 2013; Ariel et al., 2015); for example, antisense transcripts control sense transcriptional levels, splicing, RNA stability and translational efficiency. (Lyle et al., 2000; Beltran et al., 2008; Faghihi et al., 2008; Swiezewski et al., 2009; Carrieri et al., 2012; Sarkar et al., 2015). As for their other functions, antisense transcripts participate in generation of natural antisense short interfering RNA (nat-siRNA) with sense transcriptions (Borsani et al., 2005; Ron et al., 2010). It remains unknown whether aberrant antisense non-coding RNA has biological functions. We hypothesized that aberrant antisense non-coding RNA would be subject to decapping in the wild-type, and that these non-functional transcripts are present in relatively low levels compared with protein-coding transcripts. In addition, it is unclear how functional and junk non-coding transcripts are distinguished by the RNA metabolic machinery. Among the antisense transcripts, capped and uncapped transcripts are present in *Arabidopsis* (Okamoto and Seki, 2011). *COOLAIR* transcripts that target *FLOWERING LOCUS C* (*FLC*), which encodes a floral regulator in *Arabidopsis*, control *FLC* silencing via chromatin modification during vernalization (Swiezewski et al., 2009). An antisense transcript to mouse *ubiquitin carboxy-terminal hydrolase L1* (*Uchl1*), which is involved in neurodegenerative diseases, increases the translation of *Uchl1* (Carrieri et al., 2012). These are known as the functional antisense version of long non-coding RNA, has both a cap and a poly (A) tail (Swiezewski et al., 2009; Carrieri et al., 2012). By contrast, *Xist activating RNA* (*XistAR*), which is an antisense long non-coding RNA to *Xist* in mouse, has cap structure at the 5′ end of, but no poly-A+ tail at the 3′ end of the transcript (Sarkar et al., 2015). In addition, *COLDAIR*, which is a sense long non-coding RNA to *FLC*, also has a cap structure at the 5′ end of the transcripts, but no poly-A+ tail (Heo and Sung, 2011). These observations suggest that antisense transcripts having a cap structure might be one of the features of functional non-coding transcripts. Profiling mRNA 5′ ends and strand specific transcriptome analysis using next-generation sequencing will help to identify functional antisense transcripts.

Although we have identified non-AGI transcripts using a strand specific tiling array, their actual size and 3′ terminus structures were not determined in this study. However, it is probable that these transcripts will have poly(A) tails at their 3′ termini, because an oligo(dT) primer was used for probe synthesis for the tiling array analysis. Among the identified non-AGI TUs, 198 TUs are registered as AGI-code genes, and 53 out of 198 TUs are antisense AGI-code gene to the opposite strand of a previously reported AGI-code in the latest version of TAIR 10 (Supplementary Table S3). Nevertheless, to characterize aberrant transcripts fully, isolation of full-length cDNAs will be required, regardless of whether they have a cap structure and poly (A) tail at their 5′ and 3′ termini.

## Author Contributions

MO and MS designed and interpreted the experiments; MO, MT, and JI conducted the experiments; MO, AM, TM, KI, YM, and TT analyzed the data; MO and MS wrote the paper.

## Conflict of Interest Statement

The authors declare that the research was conducted in the absence of any commercial or financial relationships that could be construed as a potential conflict of interest.

## Footnotes

1 http://www.ncbi.nlm.nih.gov/geo/info/linking.html

## Supplementary Material

Figure S1 **Complementation of the *lsm5/sad1* mutant by the *LSM5/SAD1* gene. (A)** Photographs of wild-type, *lsm5/sad1* mutant and promoter_LSM5/SAD1_::LSM5/SAD1-sGFP in *lsm5/sad1* transformant (#1 and #2) plants after heat stress. **(B)** The survival rate of heat-treated plants after heat stress at the temperatures indicated. The values are means ± SEM of the result from five plates (∼25 plants per plate) of individual lines. **(C)** Chlorophyll content of heat-treated plants. **(B,C)** The values are means ± SEM of the result from five experiments of individual lines. **(A**-**C)** Five-day-old seedlings of the wild-type, *lsm5/sad1* mutant and T3 transformant were subjected to 43 or 44°C for 90 min, returned to 22°C under the light, and the photographs, survival rate and chlorophyll contents were obtained after 9 days of growth.
