# Supplementary figures and images for: Sm-Like Protein-Mediated RNA Metabolism Is Required for Heat Stress Tolerance in Arabidopsis
Source: Front Plant Sci. 2016 Jul 21;7:1079. doi: 10.3389/fpls.2016.01079 (PMC4954817; doi:10.3389/fpls.2016.01079)

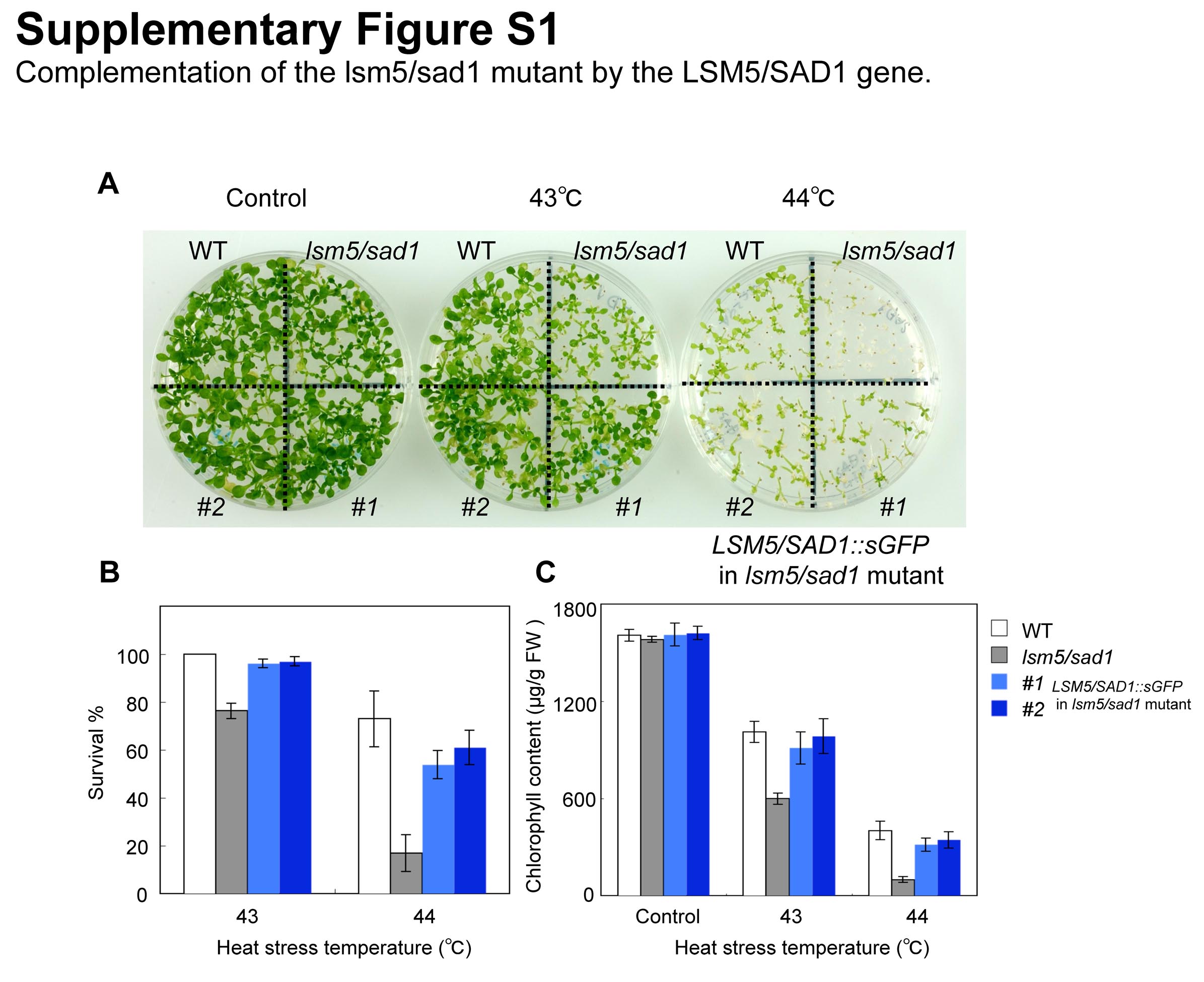

Supplement: Figure S1 — Complementation of the lsm5/sad1 mutant by the LSM5/SAD1 gene. (A) Photographs of wild-type, lsm5/sad1 mutant and promoterLSM5/SAD1::LSM5/SAD1-sGFP in lsm5/sad1 transformant (#1 and #2) plants after heat stress. (B) The survival rate of heat-treated plants after heat stress at the temperatures indicated. The values are means ± SEM of the result from five plates (∼25 plants per plate) of individual lines. (C) Chlorophyll content of heat-treated plants. (B,C) The values are means ± SEM of the result from five experiments of individual lines. (A-C) Five-day-old seedlings of the wild-type, lsm5/sad1 mutant and T3 transformant were subjected to 43 or 44°C for 90 min, returned to 22°C under the light, and the photographs, survival rate and chlorophyll contents were obtained after 9 days of growth. [file Image_1.JPG]
